# Identification of candidate blood biomarkers through metabolomics analysis in bovine superovulation

**DOI:** 10.3389/fvets.2025.1552045

**Published:** 2025-04-30

**Authors:** Xiaohu Su, Guangqi Gao, Liqiang Chen, Liguo Zhang, Guangnan Liu, Chao Bian, Guanghua Su, Lei Yang

**Affiliations:** ^1^State Key Laboratory of Reproductive Regulation and Breeding of Grassland Livestock, School of Life Sciences, Inner Mongolia University, Hohhot, Inner Mongolia Autonomous Region, China; ^2^Key Laboratory of Dairy Products Processing, Ministry of Agriculture and Rural Affairs, Inner Mongolia Agricultural University, Hohhot, Inner Mongolia Autonomous Region, China; ^3^Ulanqab Animal Husbandry Workstation, Ulanqab Agriculture and Animal Husbandry Bureau, Ulanqab, Inner Mongolia Autonomous Region, China; ^4^Tumor Radiotherapy Department, Inner Mongolia People’s Hospital, Hohhot, Inner Mongolia Autonomous Region, China

**Keywords:** cow, superovulation, metabolome, serum, biomarker

## Abstract

Superovulation and embryo transfer technologies provide strong support for improving the productivity of cattle population. A non-invasive diagnostic method for superovulation prediction is necessary to improve its efficiency. Compared to macromolecular substances, there has been an increasing number of studies on small molecular metabolites as biomarkers. This study aimed to identify key biomarkers associated with superovulation outcomes in cows through serum metabolomics analysis. In this study, 36 induced estrus cows were selected, and the blood samples were collected at three time points: before FSH injection, before artificial insemination, and before embryo collection. Then, the cows were classified into high embryonic yield (HEY) and low embryonic yield (LEY) groups based on the total number of embryos. Furthermore, a serum untargeted metabolomics analysis of the two groups was conducted using liquid chromatography with tandem mass spectrometry (LC–MS/MS). A total of 372 embryos were collected. The metabolomics analysis revealed that 1,158 metabolites were detected, and 617 were annotated. In the before FSH injection samples, 121 differential metabolites were identified between the two groups. In the before artificial insemination samples, 129 differential metabolites were identified. In the before embryo collection samples, 201 differential metabolites were identified. A total of 11 differential metabolites were shared between the before FSH injection and before artificial insemination samples, while five differential metabolites were shared across all three samples. The majority of the differential metabolites were significantly enriched in pathways related to amino acid and fatty acid metabolism, digestive system secretion, and ovarian steroidogenesis. This study showed that phosphatidylcholine [PC; 14:0/22:1(13Z)], phosphatidylethanolamine [PE; DiMe (11, 3)], triacylglycerol [TG; 15:0/16:0/22:4 (7Z, 10Z, 13Z, 16Z)], phosphatidylinositol [PI; 16:0/22:2 (13Z, 16Z)], and phosphatidylserine [PS; 18:0/20:4(8Z, 11Z, 14Z, 17Z)] were differentially expressed in the serum during the superovulation period. These could serve as potential biomarkers for embryonic yield prediction in bovine superovulation. The lipid and amino acid metabolic pathways may have an impact on the ovarian response. The results of this study could provide novel screening indexes of donors for bovine superovulation, although the accuracy of the relevant factors requires further investigation.

## Introduction

The cattle industry is an important part of animal husbandry. Superovulation and embryo transfer technologies provide powerful support for improving the productivity of cattle population. However, the efficiency of bovine superovulation is affected by multiple factors, including genetics, age, body condition, diseases, and management, likely through their influence on the uterine environment, oocyte quality, and embryo development (1). To improve the screening efficiency of donors for bovine superovulation, several strategies have been established, including small follicle detection by ultrasound (2), genotype analysis (3–5), reproductive hormone detection (AMH, P4, E2, and FSH) (6–13), blood vitamin levels (14), paraoxonase-1 (15), serum biochemical parameters (16), and partial metabolic parameters (17, 18). However, the ovarian antral follicle count does not have a linear correlation with the outcome of embryo production (19). The prediction efficiency of reproductive hormone levels varies with age (11). In addition, the detection time is also important. Compared to macromolecular substances, there have been an increasing number of studies on small molecule metabolites as biomarkers.

Metabolites are the end products of various biochemical reactions in cells. Their composition and activity are influenced by both internal and external factors. Many studies have been conducted on the metabolome and bovine reproduction, including reproductive performance (17, 18, 20), pregnancy prediction (21–24), and reproductive disorders (1, 25–27). In the research by Horn et al., the preovulatory serum metabolome was analyzed in cows with different body condition scores to explain the mechanisms by which extreme body conditions affect reproductive capacity (18). However, more studies are necessary to identify reliable metabolic biomarkers related to bovine superovulation and to more comprehensively reveal the regulatory mechanisms, as metabolites are affected by many factors.

In this study, differences in serum reproductive hormones and metabolites between high embryonic yield (HEY) and low embryonic yield (LEY) HuaXi cows were analyzed during the superovulation period to identify key biomarkers related to superovulation outcomes. The study results may provide novel screening indexes of donors for bovine superovulation.

## Results

### Embryo production

**Figure 1:**
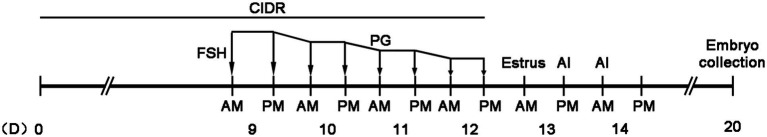
CIDR (Zoetis, New Zealand) was inserted into the vagina on day 0 (D0). Between days 9 and 12, a total of 500 μg of FSH (Stimufol^®^, Belgium) was injected in eight doses, with each dose decreasing by 10%. On the fifth FSH injection (morning of D11), 300 μg of PG (Reprobiol, New Zealand) was injected synchronously. The CIDR was removed at the time of the final FSH injection. Estrus detection occurred on D13, and AI was performed twice (12 h interval). On D20, embryos were collected via uterus flushing after the cows were anesthetized with a caudal spinal injection of lidocaine hydrochloride.

In this study, 36 cows were selected and treated using the standard superovulation process (Figure 1). The results are shown in Table 1. A total of 372 embryos were collected, with an average of 10.33 ± 1.25 embryos per cow. Of these, 286 embryos were viable, with an average of 7.94 ± 1.16 embryos per cow. Finally, 11 cows were assigned to the HEY group, while 9 cows were assigned to the LEY group.

**Table 1:** Statistics of embryonic production after superovulation in cows (mean ± S.D.).

Donor number	Total embryos	Average total embryos	Total viable embryos	Average viable embryos	HEY donor number	Average total embryos of HEY	Average viable embryos of HEY	LEY donor number	Average total embryos of LEY	Average viable embryos of LEY
36	372	10.33 ± 1.25	286	7.94 ± 1.16	11	18.64 ± 2.15	14.91 ± 2.35	9	2.78 ± 0.46	2.22 ± 0.36

### Untargeted metabolic profiling of the cows’ serum during superovulation

To explore the serum metabolic differences between HEY and LEY groups, untargeted metabolomics analysis was performed. In total, 13,222 features were detected, of which 5,654 metabolites were annotated (Supplementary Table S1). According to the Human Metabolome Database (HMDB) analysis, the most enriched category was lipids and lipid-like molecules (353 metabolites), followed by organic acids and derivatives (75 metabolites) and benzenoids (69 metabolites; Supplementary Figure S1A). According to the Kyoto Encyclopedia of Genes and Genomes (KEGG) analysis, the most enriched pathway was metabolic pathways (206 metabolites), followed by glycerophospholipid metabolism (135 metabolites) and the biosynthesis of secondary metabolites (75 metabolites; Supplementary Figure S1B).

### Multivariate statistical analysis

**Figure 2:**
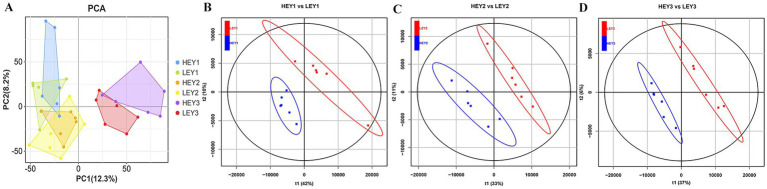
Multivariate statistical analysis results of the metabolome. **(A)** PCA results. B–D: OPLS-DA results from three sampling time points: **(B)** before FSH injection; **(C)** before artificial insemination; and **(D)** before embryo collection. HEY1: High embryonic yield group at the sampling before FSH injection. LEY1: Low embryonic yield group at the sampling before FSH injection. HEY2: High embryonic yield group at the sampling before artificial insemination. LEY2: Low embryonic yield group at the sampling before artificial insemination. HEY3: High embryonic yield group at the sampling before embryo collection. LEY3: Low embryonic yield group at the sampling before embryo collection.

Principal component analysis (PCA) was conducted to determine the separation and aggregation of samples between HEY and LEY groups. The first principal component (PC1) and the second principal component (PC2) were 12.3 and 8.2%, respectively (Figure 2A). Orthogonal partial least squares discriminant analysis (OPLS-DA) was performed to further investigate the variables responsible for classification and to achieve better group separation. For the before FSH injection samples, the R2 of the OPLS-DA model was 0.996 and the Q2 was 0.736 (Figure 2B). In the before artificial insemination samples, the R2 and Q2 were 0.996 and 0.796, respectively (Figure 2C). In the before embryo collection samples, the R2 and Q2 were 0.988 and 0.553, respectively (Figure 2D). The results showed that serum metabolites could be used to distinguish between the two groups.

### Identification of differential metabolites

The liquid chromatography with tandem mass spectrometry (LC–MS/MS) data were used to analyze the metabolites of different substances. Differential metabolites were screened based on the following criteria: VIP ≥ 1, FC > 1.2 or < 0.83, and *p* ≤ 0.05. A comprehensive statistical analysis was performed.

**Figure 3:**
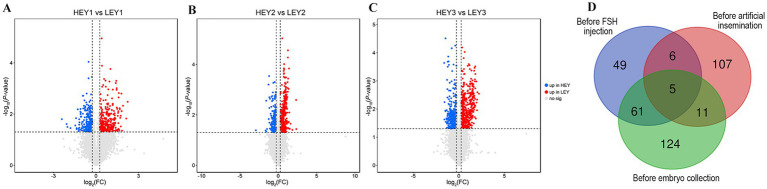
Differential metabolite analysis results. **(A–C)** Volcano plots for three sampling time points: **(A)** Before FSH injection; **(B)** before artificial insemination; and **(C)** before embryo collection. **(D)** Venn diagram of the differential metabolites across three sampling time points. HEY1: High embryonic yield group at the sampling before FSH injection. LEY1: Low embryonic yield group at the sampling before FSH injection. HEY2: High embryonic yield group at the sampling before artificial insemination. LEY2: Low embryonic yield group at the sampling before artificial insemination. HEY3: High embryonic yield group at the sampling before embryo collection. LEY3: Low embryonic yield group at the sampling before embryo collection.

In the before FSH injection samples, 121 differential metabolites were identified, of which 23 were upregulated in the HEY group and 98 were upregulated in the LEY group (Figure 3A; Supplementary Table S2). In the before artificial insemination samples, 129 differential metabolites were identified, of which 28 were upregulated in the HEY group and 101 were upregulated in the LEY group (Figure 3B; Supplementary Table S3). In the before embryo collection samples, 201 differential metabolites were identified, of which 53 were upregulated in the HEY group and 148 were upregulated in the LEY group (Figure 3C; Supplementary Table S4).

Among the differential metabolites at three sampling time points, there were 11, 16, and 66 shared differential metabolites between the before FSH injection and before artificial insemination samples, the before artificial insemination and before embryo collection samples, and the before FSH injection and before embryo collection samples, respectively (Figure. 3D; Supplementary Tables S5–S7). In addition, five shared differential metabolites were identified across all three sampling time points. Among them, phosphatidylcholine [PC; 14:0/22:1(13Z)] was upregulated in the HEY group, while phosphatidylethanolamine [PE; DiMe (11, 3)], triacylglycerol [TG; 15:0/16:0/22:4 (7Z, 10Z, 13Z, 16Z)], phosphatidylinositol [PI; 16:0/22:2 (13Z, 16Z)], and phosphatidylserine [PS; 18:0/20:4(8Z, 11Z, 14Z, 17Z)] were upregulated in the LEY group at all three time points (Figure 3D; Table 2).

**Table 2:** Shared differential metabolites across three sampling time points.

Metabolite ID	MS1 name	MZ	Sampling time	Relative abundance of HEY group	Relative abundance of LEY group	Fold change^a^	*p* value^b^	VIP^c^	Up. down^d^
POS_788.6168_5.679	PC[14:0/22:1(13Z)]	788.62	Before FSH injection	1661555.38	1179292.10	1.41	0.0220	9.05	up
			Before artificial insemination	1940812.82	1320729.14	1.47	0.0222	8.94	up
			Before embryo collection	2104757.61	1350278.94	1.56	0.0240	8.33	up
NEG_811.5229_7.6312	PE [DiMe(11,3)]	811.52	Before FSH injection	19015.44	52112.66	0.36	0.0011	2.38	down
			Before artificial insemination	31786.16	61470.94	0.52	0.0347	1.88	down
			Before embryo collection	16952.03	52399.75	0.32	0.0036	2.11	down
NEG_884.7788_7.6502	TG[15:0/16:0/22:4(7Z,10Z,13Z,16Z)]	884.78	Before FSH injection	11372.16	21573.02	0.53	0.0315	1.25	down
			Before artificial insemination	15427.56	37777.81	0.41	0.0332	1.78	down
			Before embryo collection	9420.85	25223.36	0.37	0.0025	1.40	down
NEG_889.575_7.8226	PI[16:0/22:2(13Z,16Z)]	889.57	Before FSH injection	533499.10	1606997.01	0.33	0.0022	14.04	down
			Before artificial insemination	907624.75	2356853.26	0.39	0.0348	12.79	down
			Before embryo collection	482513.64	1404412.95	0.34	0.0060	10.55	down
NEG_810.5216_7.6216	PS[18:0/20:4(8Z,11Z,14Z,17Z)]	810.52	Before FSH injection	55926.94	147153.83	0.38	0.0006	4.07	down
			Before artificial insemination	85709.53	170426.08	0.50	0.0446	3.14	down
			Before embryo collection	51423.13	152406.47	0.34	0.0037	3.57	down

a The fold change between the high embryonic yield group and the low embryonic yield group (a higher ratio indicates a higher level of expression of a compound in the high embryonic yield group).

b *p-value represents the significance level of the difference between the two groups.*

c Variable importance in projection of the two groups.

d Compared to the low embryonic yield group, the high embryonic yield group showed upregulated or downregulated expression of this metabolite.

### Functional enrichment of the differential metabolites

**Figure 4:**
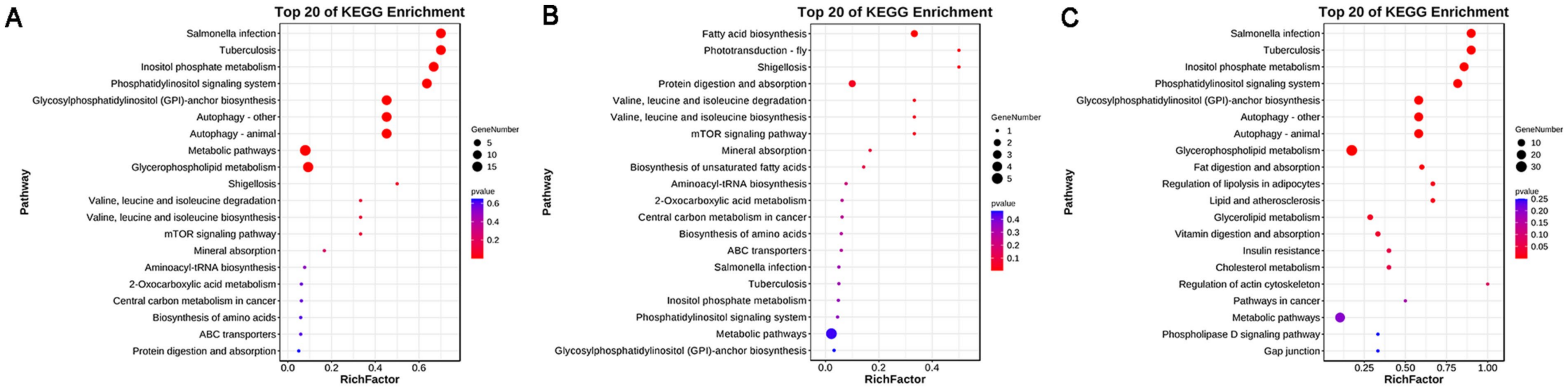
KEGG enrichment results of differential metabolites. **(A)** Before FSH injection; **(B)** before artificial insemination; **(C)** before embryo collection.

The functional enrichment of differential metabolites was performed following the method described by Kanehisa et al. (28). The results showed that 21 pathways were enriched in the before FSH injection samples. Among these, nine pathways were significantly enriched, including inositol phosphate metabolism, phosphatidylinositol signaling system, and glycosylphosphatidylinositol (GPI)-anchored biosynthesis, among others (Figure 4A). A total of 24 pathways were enriched in the before artificial insemination samples. Among these, three pathways were significantly enriched, including fatty acid biosynthesis, phototransduction–fly, and shigellosis (Figure 4B). A total of 24 pathways were enriched in the before embryo collection samples. Among these, 13 pathways were significantly enriched, including inositol phosphate metabolism, fat digestion and absorption, and regulation of lipolysis in adipocytes, among others (Figure 4C).

## Discussion

In bovine superovulation, the physiological status of the donor plays an important role in the outcome. Metabolites in the blood can reflect the physiological status of the body (29). Here, we found that there were distinct serum metabolites between bovine donors with different embryo yields at the time points before FSH injection, before artificial insemination, and before embryo collection. Phosphatidylcholine [PC; 14:0/22:1(13Z)], phosphatidylethanolamine [PE; DiMe (11, 3)], triacylglycerol [TG; 15:0/16:0/22:4 (7Z, 10Z, 13Z, 16Z)], phosphatidylinositol [PI; 16:0/22:2 (13Z, 16Z)], and phosphatidylserine [PS; 18:0/20:4(8Z, 11Z, 14Z, 17Z)] were the shared differential metabolites. The functions of these differential metabolites include fatty acid and protein metabolism and GPI-anchor biosynthesis, among others.

Several studies have shown that lipids present in follicular fluid are associated with the development of oocyte, and the concentration of lipids in the blood can reflect the concentration of follicular fluid. Furthermore, obesity is associated with irregular reproductive cycles. In obese horses, oocytes had lower concentrations of lipids consistent with PC and PE, while lipids consistent with TG tended to be higher (30). In patients with polycystic ovary syndrome, lipid profiling of follicular fluid showed differences in PC, PS, PI, and PE, strongly suggesting that these lipids may serve as biomarkers for pregnancy outcomes (31). In a study comparing human lipid profiles of follicular fluid between young poor ovarian responders and normal responders, lipids related to PC, PE, and PI were differentially expressed. These lipids may be involved in hormonal responses and oocyte development processes (32). PE, a glycerophospholipid, plays a role in the biosynthesis of PC (33). In this study, PE-NMe(11D3/11D3) was identified as a differential metabolite and may serve as a biomarker for the ovarian response during bovine ovulation.

A study on PC in bovine follicular fluid found variations during superovulation and estrous synchronization treatments, indicating that superovulation could increase the phospholipid content in follicular fluid (34). Additionally, a human superovulation study showed that some PC-related lipids in follicular fluid had higher concentrations in the successful group (32). In an ovine superovulation study, we found that certain PC-related metabolites in donor serum were significantly different between HEY and LEY populations (35). In this study, we found that PC [14:0/22:1(13Z)] was significantly higher in the HEY group than in the LEY group at all three time points. PC [14:0/22:1(13Z)] is a glycerophospholipid in which a PC moiety occupies the glycerol substitution site. Phospholipids are ubiquitous in nature and are key components of the lipid bilayer in cells. They also play crucial roles in metabolism and signaling (36). Taken together, PC [14:0/22:1(13Z)] may serve as a serum biomarker for bovine superovulation.

Lipid metabolism provides the energy source during oocyte maturation. Lipids in oocytes are primarily triglycerides composed of specific fatty acids, which differ by species (37). In cumulus–oocyte complexes (COCs), TG are metabolized by lipases and through *β*-oxidation in mitochondria for ATP production (37). In dairy cows, follicular fluid TG levels were lower than those in serum, but there was a significant correlation between the two (38). In humans, studies have indicated that the majority of serum metabolites, including TG, are also present in follicular fluid, albeit at reduced levels (39). In adult zebrafish, high blood TG levels had negative effects on their offspring (40). Interestingly, Calonge et al. (41) found that the lipid profiles in follicular fluid and plasma inversely and significantly influenced ovarian response and the number of matured oocytes recovered. In this study, we found that TG composition [15:0/16:0/22:4(7Z,10Z,13Z,16Z)] was higher in the LEY group comparted to the HEY group. This suggests that excessively high blood TG levels affect the ovarian response during bovine superovulation.

PI could help maintain the low activity levels of the Hippo pathway (42). Emerging studies have discovered that the Hippo pathway plays an important role in regulating ovarian physiology and fertility (43). The process of oocyte maturation depends on follicle development. Previous studies have demonstrated that the Hippo pathway regulates the activation and growth of follicles (44–47). Related research has suggested that the upstream Hippo component, SAV1/MST1/2, contributes to the suppression of ovarian granulosa cell proliferation, while the Hippo effector YAP1 is essential for the proliferation of these granulosa cells (48). In summary, the serum metabolites related to PI may regulate follicle development through the Hippo pathway. In this study, we found that the level of PI [16:0/22:2 (13Z, 16Z)] was significantly higher in the HEY group than in the LEY group at all three sampling time points. This finding may be associated with the outcome of embryo production.

## Conclusion

Serum metabolomics analysis provides a feasible approach to explore the predictors of superovulation in bovine. This study showed that PC [14:0/22:1(13Z)], PE [DiMe (11, 3)], TG [15:0/16:0/22:4 (7Z, 10Z, 13Z, 16Z)], PI [16:0/22:2 (13Z, 16Z)], and PS [18:0/20:4(8Z, 11Z, 14Z, 17Z)] were differentially expressed in the serum during the superovulation period. These could serve as potential biomarkers for embryonic yield prediction in bovine superovulation. The lipid and amino acid metabolic pathways may have an impact on the ovarian response. The study results may provide novel screening indexes of donors for bovine superovulation, although the accuracy of the relevant factors requires further investigation.

## Methods

### Experimental location and cows’ management

This study was conducted at the Livestock Experimental Base of Inner Mongolia University in Hohhot (Inner Mongolia, China). This region has a tropical mid-temperate, semi-arid continental monsoon climate, characterized by long dry winters and short rainy summers.

A total of 36 sexually mature and clinically healthy Huaxi cows, aged 2 to 4 years, were used in this study. The cows’ diet was designed to meet their nutritional needs, with unified management during feeding in the barn. The cows were fed a total mixed ration consisting of 15.00% alfalfa hay, 50.00% whole corn silage, and 35.00% cow concentrate supplement, resulting in a forage-to-concentrate ratio of 65:35.

### Superovulation protocols

The cows were treated as follows (Figure 1): A CIDR (Zoetis, New Zealand) was inserted into the vagina on day 0 (D0). Between days 9 and 12, a total of 500 μg of FSH (Stimufol^®^, Belgium) was injected in eight doses, with each dose decreasing by 10%. On the fifth FSH injection (morning of D11), 300 μg of PG (Reprobiol, New Zealand) was injected synchronously. The CIDR was removed at the time of the final FSH injection. Estrus detection occurred on D13, and artificial insemination (AI) was performed twice (12 h interval). On D20, embryos were collected via uterus flushing after the cows were anesthetized with a caudal spinal injection of lidocaine hydrochloride.

### Blood sampling

Blood samples were collected in the mornings on D9, before FSH injection; on D13, before artificial insemination; and on D20, before embryo collection. The samples were collected in vacuum blood collection tubes. Then, the samples were centrifuged at 10000 × g for 10 min to obtain the serum. All serum samples were stored in liquid nitrogen until further use.

### LC–MS/MS measurements for serum metabolomics

Based on the total embryonic yield, the cows with ≥ 13 embryos were classified into the high embryonic yield (HEY) group, and the cows with ≤ 5 embryos were classified into the low embryonic yield (LEY) group. Six cows from each group (*n* = 6) were selected for serum metabolomics analysis. The LC–MS/MS-based serum metabolomics analysis was performed by LC-Bio Co. Ltd. (Hangzhou, China), following the methodology described by Xu et al. (35).

Briefly, the serum samples were purified with methanol and diluted in LC–MS grade water. UHPLC–MS/MS analyses were performed using a Vanquish UHPLC system (Thermo Fisher Scientific, Germany) coupled with an Orbitrap Q Exactive^TM^ HF mass spectrometer (Thermo Fisher Scientific, Germany). The solvent gradient was set as follows: 2% B, 1.5 min; 2–85% B, 3 min; 100% B, 10 min; 100–2% B, 10.1 min; and 2% B, 12 min. The Q Exactive^TM^ HF mass spectrometer was operated in both positive and negative polarity modes, with a spray voltage of 3.5 kV, a capillary temperature of 320°C, a sheath gas flow rate of 35 psi, an auxiliary gas flow rate of 10 L/min, an S-lens RF level of 60, and an auxiliary gas heater temperature of 350°C.

### Data processing and metabolite identification

Briefly, raw data files generated through UHPLC–MS/MS were processed using Compound Discoverer 3.1 (CD3.1, Thermo Fisher Scientific, Germany). Then, the peaks were matched with mzCloud,^1^ mzVault, and MassList databases to obtain accurate qualitative and relative quantitative results. Statistical analyses were performed using R statistical software (version R-3.4.3), Python (Python 2.7.6 version), and CentOS (CentOS release 6.6).

### Data analysis

The Kyoto Encyclopedia of Genes and Genomes (KEGG),^2^ the LIPID MAPS (Lipidmaps),^3^ and the Human Metabolome Database (HMDB)^4^ were used to annotate the biological functions of the metabolites. Bioinformatic analysis was performed using R software (v.4.0.2) or the OmicStudio tools available at https://www.omicstudio.cn/tool. The metabolism data were statistically analyzed using the two-sided Wilcoxon rank sum test and finally visualized using the ggpubr package (version 0.6.0). The metabolites with a variable importance in projection (VIP) > 1, a *p*-value < 0.05, and a fold change (FC) ≥ 1.2 or ≤ 0.83 were considered differential metabolites. Volcano plots were used to filter the metabolites of interest based on log_2_(FoldChange) and -log_10_(*p*-value) of metabolites using ggplot2 in R language. A Venn diagram of the differential metabolites was created at http://bioinformatics.psb.ugent.be/webtools/Venn/. The functions of differential metabolites were analyzed using the KEGG database.

## Data Availability

The original contributions presented in the study are publicly available. This data can be found here: https://db.cngb.org/search/project/CNP0006676/.

## Footnotes

^1^ https://www.mzcloud.org/

^2^ http://www.kegg.com

^3^ www.lipidmaps.org

^4^ http://www.hmdb.ca

## Supplementary material

Supplementary Figure S1 Classification of cows’ serum metabolites during superovulation **(A)** Classification result of annotated metabolites by HMDB analysis. **(B)** KEGG pathway enrichment analysis result of annotated metabolites.
