# Supplementary figures and images for: Identification of candidate blood biomarkers through metabolomics analysis in bovine superovulation
Source: Front Vet Sci. 2025 Apr 30;12:1552045. doi: 10.3389/fvets.2025.1552045 (PMC12076740; doi:10.3389/fvets.2025.1552045)

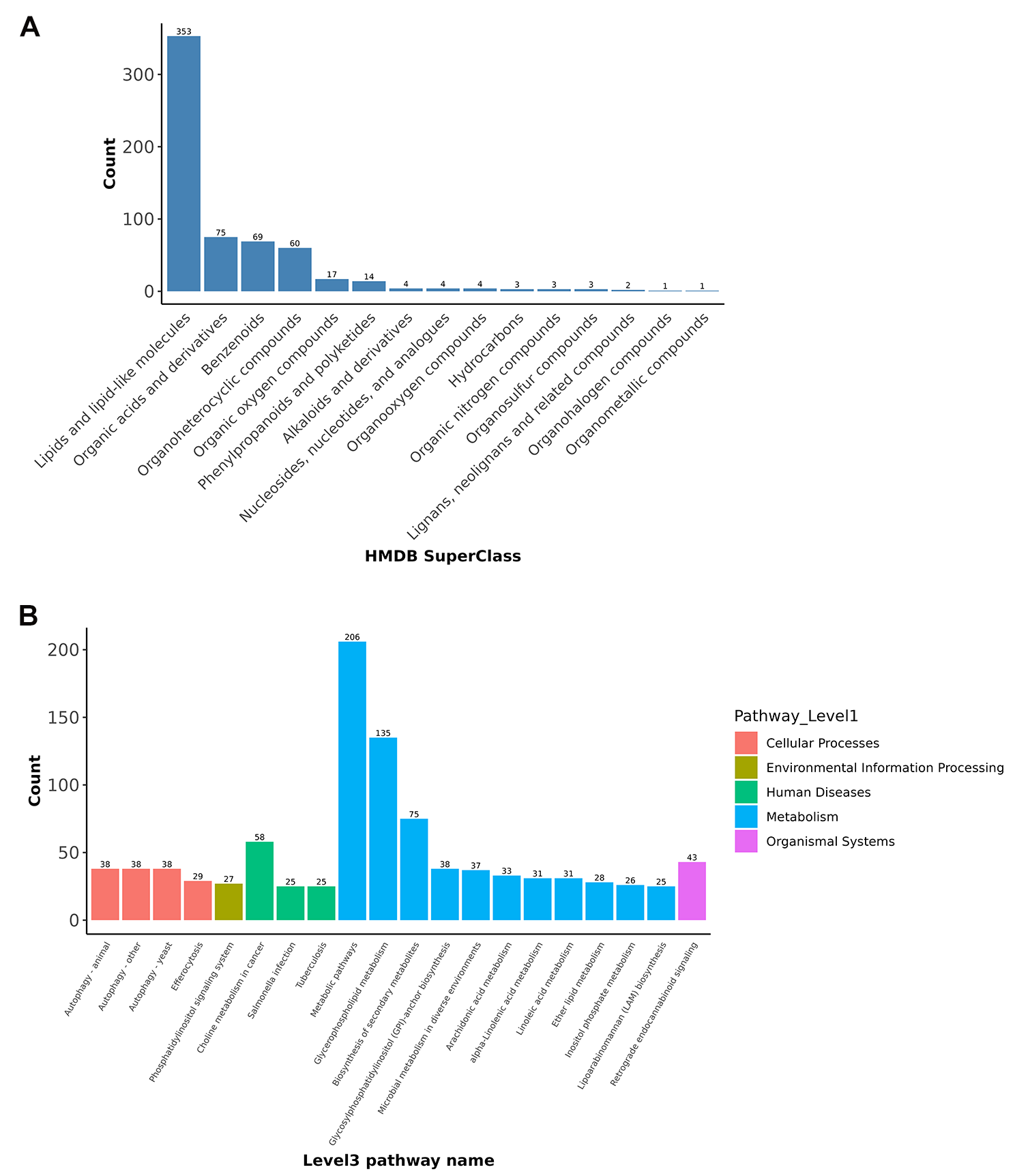

Supplement: Supplementary Figure S1 — Classification of cows’ serum metabolites during superovulation (A) Classification result of annotated metabolites by HMDB analysis. (B) KEGG pathway enrichment analysis result of annotated metabolites. [file Image_1.tif]
